# Application of a Gas-Kinetic BGK Scheme in Thermal Protection System Analysis for Hypersonic Vehicles

**DOI:** 10.3390/e24101325

**Published:** 2022-09-21

**Authors:** Di Zhou, Bingchen Du, Tongqing Guo, Qiaozhong Li, Zhiliang Lu

**Affiliations:** Key Laboratory of Unsteady Aerodynamics and Flow Control, Ministry of Industry and Information Technology, Nanjing University of Aeronautics and Astronautics, Nanjing 210016, China

**Keywords:** gas-kinetic scheme, BGK model, thermal protection system, hypersonic flow

## Abstract

One major problem in the development of hypersonic vehicles is severe aerodynamic heating; thus, the implementation of a thermal protection system is required. A numerical investigation on the reduction of aerodynamic heating using different thermal protection systems is conducted using a novel gas-kinetic BGK scheme. This method adopts a different solution strategy from the conventional computational fluid dynamics technique, and has shown a lot of benefits in the simulation of hypersonic flows. To be specific, it is established based on solving the Boltzmann equation, and the obtained gas distribution function is used to reconstruct the macroscopic solution of the flow field. Within the finite volume framework, the present BGK scheme is specially designed for the evaluation of numerical fluxes across the cell interface. Two typical thermal protection systems are investigated by using spikes and opposing jets, separately. Both their effectiveness and mechanisms to protect the body surface from heating are analyzed. The predicted distributions of pressure and heat flux, and the unique flow characteristics brought by spikes of different shapes or opposing jets of different total pressure ratios all verify the reliability and accuracy of the BGK scheme in the thermal protection system analysis.

## 1. Introduction

Increasing attention is being paid to hypersonic vehicles within the aerospace community because of their fast access to space, rapid military response at long ranges, and fast means of commercial air travel. In the long-term development of hypersonic vehicles, one of the most important problems is severe aerodynamic heating at the nose of the vehicle [1]. This makes the design and use of a thermal protection system (TPS) essential, especially for sustained long-range maneuverable flights.

Currently, many thermal protection systems have been constructed. These can be categorized into two types: active method and passive method. Active methods protect the body surface from heating by using injection gases or mechanical devices, such as evaporation cooling [2], film cooling [3], opposing jet [4], mechanical spike [5], and directed energy air spike [6]. Passive methods generally use heat protection materials [7] and ablators [8]. In the present study, active thermal protection systems are considered for their reusability and fine controllability. As the design of such TPS largely depends on the aero-thermal loads acting on the vehicle, accurate prediction of aerodynamic heating plays a vital role [9].

Hypersonic flows are usually characterized with a thin shock layer, complex wave structures, and various shock–boundary interactions. This demands higher requirements from the numerical methods. In conventional computational fluid dynamics (CFD) technology, the total fluxes across the cell interface are split into inviscid and viscous parts, and different solution strategies are adopted for them. Over the past few years, a variety of important numerical algorithms have been developed specifically to deal with inviscid fluxes. Most of them are constructed based on the mathematical and physical properties of the Euler equations and can work well for flows at moderate Mach numbers. However, they may exhibit different numerical behaviors in hypersonic flows. For instance, the JST scheme has gained popularity in aircraft design due to its lower cost, but it may encounter instabilities and accuracy degradation at a higher Mach number. The Van Leer’s flux-vector splitting scheme works very well in the case of Euler equations, but may provide inaccurate stagnation temperatures for hypersonic viscous flows. The Roe’s flux-difference splitting upwind scheme shows a high resolution in the boundary layers and a good solution for shocks, but the well-known “carbuncle” phenomenon may occur in multi-dimensional and high-speed problems. Even for the AUSM-family schemes that are very popular in hypersonic flow simulation, sometimes local pressure oscillations are found in the vicinity of shocks and in cases where the flow is aligned with the grid. Considering these defects, many improvements have been proposed to enhance the modeling capabilities, mostly through some mathematical or artificial corrections [10,11,12]. However, as has been pointed out by Xu [13], these numerical difficulties are inherently due to the deficiencies of the Euler equations at describing the realistic flow evolution process around the cell interface. Using some special modifications not only brings uncertainties and inconveniences to computations, but also covers up this inherent defect.

In the last decade, many attempts have been made to develop numerical schemes based on solving more fundamental governing equations of physics, e.g., the Boltzmann equation. Particularly, the gas-kinetic Bhatnagar–Gross–Krook (BGK) scheme [14,15] has been shown to be a promising Boltzmann-type method. Its main advantages are the following. First, using the BGK collision model gives superior dissipation characteristics, which are important for capturing flow discontinuities. Second, the BGK scheme has been proven to satisfy the entropy condition and thus avoids unphysical solutions such as the “carbuncle” phenomenon. Third, its inherent positive property ensures good robustness in low-density regions. Furthermore, from the perspective of implementation, the BGK scheme allows for calculating the total fluxes in a unified way. In a pioneering study, Xu et al. [16] proposed a multi-dimensional BGK scheme for accurately predicting viscous stress and heat flux, where flow gradients in both parallel and perpendicular directions are considered. Later, Li et al. [17] developed a BGK method with kinetic boundary conditions and applied it to the numerical study of hypersonic flow past a hollow cylinder flare model. Recently, by introducing effective relaxation time into the BGK equation, Tan et al. [18] extended the method for hypersonic turbulence simulations. Other notable works include those of Li and Fu [19], Li and Zhang [20], Yang and co-workers [21,22], to mention only a few. All of the above studies show the good prospect of the BGK scheme in hypersonic applications.

The goal of this paper is to apply the gas-kinetic BGK scheme to thermal protection system analysis, which has rarely been seen in the literatures to the best of our knowledge. This is also a further extension of the previous work [23] on the algorithm improvement of the original method. Here, two commonly used active TPSs were chosen to be studied, i.e., the spike and the opposing jet. As the implementation of both will greatly increase the complexity of hypersonic flow fields and bring a lot of new aerodynamic and aerothermal phenomena, the capabilities of the BGK scheme for hypersonic flow simulation in the presence of TPS are highlighted. It is also noted that changes in the spike configurations (spike length, shape, etc.) or opposing jet parameters (mass flow rate, total pressure ratio, etc.) could produce diverse flow fields and significantly affect the performance of the TPS; thus, the abilities of the BGK scheme to capture these characteristics were also examined.

This paper is organized as follows: Section 2 describes the BGK model for the concerned governing equations. Section 3 describes the construction and implementation of the BGK scheme. Section 4 presents the numerical results and an analysis of the typical thermal protection systems using the developed method. The last section is the conclusion.

## 2. BGK Model for the Governing Equations

Because the thermal protection systems studied in this work are axisymmetric with respect to the geometry and flow conditions, the two-dimensional (2D) axisymmetric Navier-Stokes (N-S) equations are solved, which can be written as

(1) $$\frac{\partial }{\partial t}\int_{\Omega }Wd\Omega +\frac{1}{y}\oint_{\partial \Omega }yFdS=\int_{\Omega }Qd\Omega$$

where t is time, Ω is the control volume, and y is the coordinate in the radial direction. The vectors of conservative variables W, total fluxes F, and source terms Q are given by

(2) $$\begin{array}{ccc} W=\left[\begin{array}{c} \rho \\ \rho u\\ \rho v\\ \rho E \end{array}\right], & F=\left[\begin{array}{c} \rho V\\ \rho uV+n_{x}p-n_{x}\tau_{xx}-n_{y}\tau_{xy}\\ \rho vV+n_{y}p-n_{x}\tau_{yx}-n_{y}\tau_{yy}\\ (\rho E+p)V-n_{x}\Theta_{x}-n_{y}\Theta_{y} \end{array}\right], & Q=\left[\begin{array}{c} 0\\ 0\\ p-\tau_{\theta \theta }\\ 0 \end{array}\right] \end{array}$$

where ρ, u, v, E, and p denote the density, the velocity components in the axial and radial direction, the total energy per unit mass, and the pressure, respectively. The contravariant velocity is defined as $V=n_{x}u+n_{y}v$, with $n_{x}$, $n_{y}$ being components of the unit normal vector. The notations $\tau_{xx}$, $\tau_{xy}$, $\tau_{yx}$, $\tau_{yy}$, $\tau_{\theta \theta }$ represent components of the viscous stress tensor and $\Theta_{x}$, $\Theta_{y}$ are the terms describing the work of the viscous stresses and heat conduction. Details of their formulations can be seen in [10].

It should be noted that the axisymmetric equations can be transformed to the 2D planar N-S equations by removing the terms related to the radius and the additional source term.

The present BGK scheme is designed to discretize the fluxes F by reconstructing the gas distribution function f at the cell interface. The time evolution of f is governed by the 2D Boltzmann equation with the BGK collision model

(3) $$\frac{\partial f}{\partial t}+\xi_{x}\frac{\partial f}{\partial x}+\xi_{y}\frac{\partial f}{\partial y}=-\frac{1}{\tau }(f-g)$$

where $\xi_{x}$ and $\xi_{y}$ denote the particle streaming velocities, τ is the collision time, and g is the equilibrium distribution function approached by f. The equilibrium state is generally assumed to be a Maxwellian distribution

(4) $$g=\rho {\left(\frac{\lambda }{\pi }\right)}^{\frac{K+2}{2}}e^{-\lambda ({\left(\xi_{x}-u\right)}^{2}+{\left(\xi_{y}-v\right)}^{2}+\zeta^{2})}$$

where λ=ρ/2p for perfect gases, K denotes the number of degrees of the internal variables ζ and is equal to 3 for diatomic gases in the 2D case, and $\zeta^{2}=\zeta_{i}\zeta_{i}$.

Because of the conservations of mass, momentum, and energy in the particle collision process, the following compatibility condition is satisfied

(5) $$\int \frac{g-f}{\tau }\psi d\Xi =0$$

where ψ is a vector of the collision invariants, defined as

(6) $$\psi ={\left[1,\xi_{x},\xi_{y},\frac{1}{2}(\xi_{x}^{2}+\xi_{y}^{2}+\zeta^{2})\right]}^{T}$$

with the notation $d\Xi =d\xi_{x}d\xi_{y}d\zeta_{1}d\zeta_{2}\cdots d\zeta_{k}$ used.

From the definition of the gas distribution function, the macroscopic mass, momentum, and energy densities of the gas flow can be written as

(7) $$W=\left[\begin{array}{c} \rho \\ \rho u\\ \rho v\\ \rho E \end{array}\right]=\int f\psi d\Xi =\int f\left[\begin{array}{c} 1\\ \xi_{x}\\ \xi_{y}\\ \frac{1}{2}(\xi_{x}^{2}+\xi_{y}^{2}+\zeta^{2}) \end{array}\right]d\Xi$$

## 3. Gas-Kinetic BGK Scheme

### 3.1. Solution of the BGK Equation

It can be proven from the Chapman–Enskog expansion that the above BGK model recovers the governing axisymmetric N-S equations, which serves as the theoretical basis for the present BGK scheme. By using the method of characteristics, the generalized solution of Equation (3) at any time t and any cell interface $x_{i+1/2}$ is

(8) $$f(x_{i+1/2},t,\xi ,\zeta )=\frac{1}{\tau }\int_{0}^{t}e^{-(t-t^{\prime })/\tau }g(x(t^{\prime }),t^{\prime },\xi ,\zeta )dt^{\prime }+e^{-t/\tau }f_{0}(x(0),0,\xi ,\zeta )$$

where $x(t^{\prime })=x_{i+1/2}-(t-t^{\prime })\xi$ describes a particle motion trajectory with $t^{\prime }\in [0,t]$ and $\xi ={\left[\xi_{x},\xi_{y}\right]}^{T}$. The solution f describes the gas evolution process, which starts with an initial state $f_{0}$ and approaches its equilibrium state g. In the following, we show how to determine these two unknowns. For simplicity, the x—direction and y—direction are assumed as the normal and tangential directions to the local cell interface, respectively, and $x_{i+1/2}={\left[0,0\right]}^{T}$ is assumed. Note the difference between this local coordinate system and the previous global axial–radial system.

First, we construct the initial state $f_{0}$. To account for the flow discontinuities, which are common in hypersonic flows, both equilibrium and non-equilibrium distribution functions should be considered. The second-order accuracy is constructed as

(9) $$f_{0}(x(0),\xi ,\zeta )=\{\begin{array}{l} g^{l}\left[1-(a^{l}\cdot \xi )t-\tau (A^{l}+a^{l}\cdot \xi )\right],\begin{array}{cc} & \;\xi_{x}>0\Leftrightarrow x<0 \end{array}\\ g^{r}\left[1-(a^{r}\cdot \xi )t-\tau (A^{r}+a^{r}\cdot \xi )\right],\begin{array}{cc} & \xi_{x}\leq 0\Leftrightarrow x\geq 0 \end{array} \end{array}$$

where $g^{l}$ and $g^{r}$ denote the local Maxwellians defined at the left and right sides of the cell interface, respectively, and the corresponding slopes $a^{l(r)}={\left[a^{l(r)},b^{l(r)}\right]}^{T}$ and $A^{l(r)}$ are related to the spatial and temporal derivatives of $g^{l(r)}$, respectively

(10) $$\begin{array}{l} a^{l(r)}g^{l(r)}=\frac{\partial g^{l(r)}}{\partial x}\\ b^{l(r)}g^{l(r)}=\frac{\partial g^{l(r)}}{\partial y}\\ A^{l(r)}g^{l(r)}=\frac{\partial g^{l(r)}}{\partial t} \end{array}$$

The derivatives of $g^{l(r)}$ can be directly derived from Equation (4), where the left and right macroscopic states are reconstructed using the second-order MUSCL scheme. The minmod limiter is used to prevent unphysical oscillation and spurious solutions in the shock regions. By using the chain rule for the derivatives in Equation (10) and rearranging the terms, the spatial and temporal slopes can be expressed as a linear combination of the collision invariants

(11) $$\begin{array}{cc} \begin{array}{c} a^{l(r)}=a_{\alpha }^{l(r)}\psi_{\alpha }\\ b^{l(r)}=b_{\alpha }^{l(r)}\psi_{\alpha }\\ A^{l(r)}=A_{\alpha }^{l(r)}\psi_{\alpha } \end{array}, & \alpha =1,2,3,4 \end{array}$$

where $a_{\alpha }^{l(r)}$, $b_{\alpha }^{l(r)}$, and $A_{\alpha }^{l(r)}$ are local constant coefficients and have explicit formulations. For example, the expressions for $a_{\alpha }^{l(r)}$ are given by (omit the superscripts)

(12) $$\begin{array}{l} a_{1}=\frac{\partial \rho }{\rho \partial x}-2\lambda \left(u\frac{\partial u}{\partial x}+v\frac{\partial v}{\partial x}\right)+\left(\frac{K+2}{2\lambda }-u^{2}-v^{2}\right)\frac{\partial \lambda }{\partial x}\\ a_{2}=2\left(u\frac{\partial \lambda }{\partial x}+\lambda \frac{\partial u}{\partial x}\right)\\ a_{3}=2\left(v\frac{\partial \lambda }{\partial x}+\lambda \frac{\partial v}{\partial x}\right)\\ a_{4}=-2\frac{\partial \lambda }{\partial x} \end{array}$$

The same holds for $b_{\alpha }^{l(r)}$, only by changing ∂/∂x to ∂/∂y. The flow gradients in the above formulations are obtained by applying Green’s theorem to the respective cells. As for $A_{\alpha }^{l(r)}$, because the non-equilibrium part of $f_{0}$ does not directly contribute to conservative variables, thus we have

(13) $$A_{\alpha }^{l(r)}\int g^{l(r)}\psi_{\alpha }\psi d\Xi =-\int g^{l(r)}(a^{l(r)}\xi_{x}+b^{l(r)}\xi_{y})\psi d\Xi$$

from which $A_{\alpha }^{l(r)}$ can be solved.

Next we construct the time-dependent equilibrium state g shown in Equation (8). Also to the second-order accuracy, it can be expressed as

(14) $$g(x(t^{\prime }),t^{\prime },\xi ,\zeta )=\{\begin{array}{l} g_{0}\left[1+\bar{A}t^{\prime }-{\bar{a}}^{l}\xi_{x}(t-t^{\prime })-{\bar{b}}^{l}\xi_{y}(t-t^{\prime })\right],\begin{array}{cc} & \xi_{x}>0\Leftrightarrow x<0 \end{array}\\ g_{0}\left[1+\bar{A}t^{\prime }-{\bar{a}}^{r}\xi_{x}(t-t^{\prime })-{\bar{b}}^{r}\xi_{y}(t-t^{\prime })\right],\begin{array}{cc} & \xi_{x}\leq 0\Leftrightarrow x\geq 0 \end{array} \end{array}$$

where $g_{0}$ is an initial Maxwellian. The corresponding slopes ${\bar{a}}^{l(r)}={\left[{\bar{a}}^{l(r)},{\bar{b}}^{l(r)}\right]}^{T}$ and $\bar{A}$ are linked to the spatial and temporal derivatives of $g_{0}$, respectively

(15) $$\begin{array}{l} {\bar{a}}^{l(r)}g_{0}={\left(\frac{\partial g_{0}}{\partial x}\right)}^{l(r)}\\ {\bar{b}}^{l(r)}g_{0}={\left(\frac{\partial g_{0}}{\partial y}\right)}^{l(r)}\\ \bar{A}g_{0}=\frac{\partial g_{0}}{\partial t} \end{array}$$

The derivatives of $g_{0}$ are also derived from Equation (4), where the “average” macroscopic parameters $\bar{\rho }$, $\bar{\lambda }$, $\bar{u}$, $\bar{v}$ are obtained from Equations (8) and (9) with the limits x→(0,0) and $t^{\prime }\to 0$ used. This gives

(16) $$\bar{W}=\left[\begin{array}{c} \bar{\rho }\\ \bar{\rho }\bar{u}\\ \bar{\rho }\bar{v}\\ \bar{\rho }\bar{E} \end{array}\right]=\int [g^{l}H(\xi_{x})+g^{r}(1-H(\xi_{x}))]\left[\begin{array}{c} 1\\ \xi_{x}\\ \xi_{y}\\ \frac{1}{2}(\xi_{x}^{2}+\xi_{y}^{2}+\zeta^{2}) \end{array}\right]d\Xi$$

with $H(\xi_{x})$ the Heaviside function.

Then ${\bar{a}}^{l(r)}$ and ${\bar{b}}^{l(r)}$ are determined similar to Equations (11) and (12), and the gradients of “average” flow variables are obtained by applying Green’s theorem to both sides of the cell interface.

Substituting the expressions of $f_{0}$ and g into Equation (8), the generalized solution f of the BGK equation can be written as

(17) $$\begin{array}{c} f(x_{i+1/2},t,\xi ,\zeta )\\ =\gamma_{1}g_{0}+\gamma_{2}g_{0}\bar{A}+\gamma_{3}g_{0}\left[\left({\bar{a}}^{l}\cdot \xi )H(\xi_{x})+({\bar{a}}^{r}\cdot \xi )(1-H(\xi_{x})\right)\right]\\ +\gamma_{4}\left[g^{l}(1-\tau A^{l})H(\xi_{x})+g^{r}(1-\tau A^{r})(1-H(\xi_{x}))\right]\\ +\gamma_{5}\left[g^{l}(a^{l}\cdot \xi )H(\xi_{x})+g^{r}(a^{r}\cdot \xi )(1-H(\xi_{x}))\right] \end{array}$$

with the definitions of

(18) $$\begin{array}{llll} \gamma_{1}=1-e^{-t/\tau } & & & \gamma_{2}=t-\tau (1-e^{-t/\tau })\\ \gamma_{3}=te^{-t/\tau }-\tau (1-e^{-t/\tau }) & & & \gamma_{4}=e^{-t/\tau }\\ \gamma_{5}=-(t+\tau )e^{-t/\tau } & & & \end{array}$$

For the remaining unknown $\bar{A}$ in Equation (17), we can solve it using time integration of the compatibility condition (Equation (5)) over a whole time step Δt, i.e.,

(19) $$\int_{0}^{\Delta t}\int (f-g)\psi d\Xi =0$$

For the simulation of viscous flows, the collision time τ is constructed as

(20) $$\tau =\frac{\mu_{L}+\mu_{T}}{p}+c\Delta t\frac{\left|p_{l}-p_{r}\right|}{\left|p_{l}+p_{r}\right|}$$

where $\mu_{L}$ and $\mu_{T}$ denote the laminar viscosity and eddy viscosity at the cell interface, respectively. The laminar viscosity $\mu_{L}$ is calculated using the Sutherland formula, and the eddy viscosity $\mu_{T}$ is obtained by employing a turbulence model, such as the Spalart–Allmaras model [24] used here. The second term was designed for stability reasons, where c is a constant and can be chosen in the range of 1 to 5.

### 3.2. Evaluation of Numerical Fluxes

Once the time evolution of f has been obtained, the numerical fluxes at each cell interface can be evaluated according to the relations between the macroscopic variables and the microscopic distribution function, i.e.,

(21) $$F(x_{i+1/2},t)=\int \xi_{x}\psi f(x_{i+1/2},t,\xi ,\zeta )d\Xi$$

Because the original BGK model recovers the macroscopic equations with a Prandtl number of Pr=1, in order to deal with arbitrary Pr, a Prandtl number fix is used based on the modification of the energy flux. According to the definition of q, we have

(22) $$\begin{array}{c} q=\frac{1}{2}\int (\xi_{x}-\bar{u})({\left(\xi_{x}-\bar{u}\right)}^{2}+{\left(\xi_{y}-\bar{v}\right)}^{2}+\zeta^{2})fd\Xi \\ \;\;=\int \xi_{x}\left(\frac{1}{2}({\bar{u}}^{2}+{\bar{v}}^{2})\psi_{1}+\psi_{4}-u\psi_{2}-v\psi_{3}\right)fd\Xi -\bar{u}\int \left(\frac{1}{2}({\bar{u}}^{2}+{\bar{v}}^{2})\psi_{1}+\psi_{4}-\bar{u}\psi_{2}-\bar{v}\psi_{3}\right)fd\Xi \end{array}$$

By substituting the expression of f into the above equation, we obtain an accurate time-dependent q. It should be noted that all of the terms in Equation (22) were already obtained when solving the BGK equation, thus no extra moment computations are required.

Then, the Prandtl number fix is achieved by modifying the energy flux as

(23) $$F_{4}^{fix}=F_{4}+\left(\frac{1}{Pr}-1\right)q$$

For the turbulent simulations, q is divided into the laminar heat flux $q_{L}$ and turbulent heat flux $q_{T}$ according to the respective viscosities, i.e.,

(24) $$q_{L}=\frac{q\mu_{L}}{\mu_{L}+\mu_{T}},\begin{array}{cc} & q_{T}=\frac{q\mu_{T}}{\mu_{L}+\mu_{T}} \end{array}$$

and the modified energy flux becomes

(25) $$F_{4}^{fix}=F_{4}+\left(\frac{1}{Pr}-1\right)q_{L}+\left(\frac{1}{Pr_{T}}-1\right)q_{T}$$

with $Pr_{T}$ being the turbulent Prandtl number.

In the present work, a perfect gas is assumed with Prandtl numbers of Pr=0.72 and $Pr_{T}=0.9$, and a specific heat ratio of γ=1.4. The thermal conductivity coefficient k is calculated from the relationship $k=c_{p}(\mu_{L}/Pr+\mu_{T}/Pr_{T})$, with $c_{p}$ being the specific heat coefficient at a constant pressure.

### 3.3. Update of Flow Variables

It is seen from Equations (17) and (21) that both the solution f and the numerical fluxes F are time-dependent. To update the flow variables, a direct idea is to adopt explicit time integration methods such as the popular multistage schemes. However, to ensure correct flux balance throughout a cell, the computational time step $\Delta t_{c}$ should be set to be identical in the whole flow field. Moreover, for numerical stability, this can be no greater than the minimum value among all of the local time steps, i.e., $\Delta t_{c}\leq \min(\Delta t)$. Accordingly, this may significantly decrease the computational efficiency for steady-state flow computations. Instead, we adopt a more efficient approach by using a separate discretization in space and time (i.e., the method of lines), and the time-averaged fluxes are introduced as follows

(26) $$\bar{F}(x_{i+1/2})=\frac{1}{\Delta t}\int_{0}^{\Delta t}\int \xi_{x}\psi f(x_{i+1/2},t,\xi ,\zeta )d\Xi$$

where Δt is called the flux time averaging step, so as to distinguish from the computational time step $\Delta t_{c}$. With this approach, local time stepping is used to accelerate the convergence, and a non-uniform $\Delta t_{c}$ is allowed that can take a value much larger than in the original BGK scheme.

By using the cell-centered finite volume method in Equation (1) for spatial discretization, we obtain

(27) $$\frac{\partial W}{\partial t}=-\frac{1}{\Omega }\sum_{m=1}^{N_{F}}{\left(\bar{F}\Delta S\right)}_{m}+Q=R$$

where $N_{F}=4$ for structured grids, $\Delta S_{m}$ is the area of the face m, and R represents the residual vector.

To further improve the computational efficiency, the above equation is solved in an implicit way

(28) $$\left(\frac{\Omega }{\Delta t_{c}}I+\frac{\partial R}{\partial W}\right)\Delta W^{\left(n\right)}=-R^{\left(n\right)}$$

where $\Delta W^{\left(n\right)}=W^{\left(n+1\right)}-W^{\left(n\right)}$ denotes the update of the solution in time and n and n+1 are the current and new time levels, respectively. The recently-developed JFNK–BGK method [25] was employed to solve the above linearized system so as to quickly obtain an update of the flow variables. As a result, the present implicit BGK scheme has a comparable computational efficiency to conventional CFD methods.

### 3.4. Code Validation

**Figure 1:**
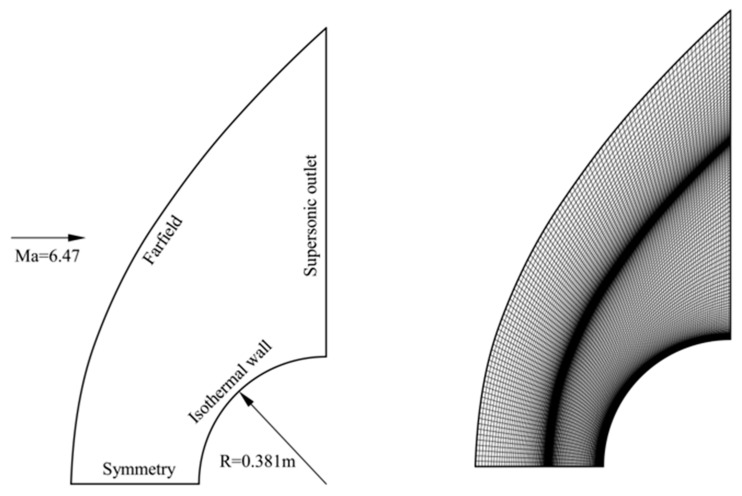
Computational model of the cylindrical leading-edge case and the grid used.

To ensure that the thermal protection analysis results obtained by the BGK scheme are reliable and accurate, validating the developed code through the simulation of “clean” hypersonic flow is essential. An example of a cylindrical leading-edge model in a Mach 6.47, Reynolds number 9.98 × 10^5^ flow (based on the model diameter) was selected to be studied. The experimental investigation of this model was conducted in the NASA Langley’s high temperature tunnel [26]. The free-stream pressure and temperature are 648.1 Pa and 241.5 K, respectively. The cylindrical surface is assumed to have a uniform temperature of *T_wall_* = 294.4. For this planar model, the computational model and the grid used are shown in Figure 1.

Different from our previous studies making assumptions about laminar flows, this work accounts for the turbulence effects. The first cell height to wall Δs is determined by the condition of $y^{+}\leq 1$. A grid sensitivity study is performed in advance, and a 101 × 201 grid (in tangential and normal directions, respectively) with Δ*s* = 1 × 10^−6^ m is selected to be used. This results in a grid-independent value of stagnation-point heat flux $q_{\mathrm{stag}}$ of 488.7 kW/m^2^. This value agrees well with those from Zhang et al. [27] (485.5 kW/m^2^), Dechaumphai et al. [28] (482.6 kW/m^2^), and our previous laminar computation [23] (488.5 kW/m^2^), although all of the experimental data are below (670.0 kW/m^2^). This discrepancy can be attributed to neglecting the 3D effects, the uncertainty in turbulence modeling, and measurement errors.

**Figure 2:**
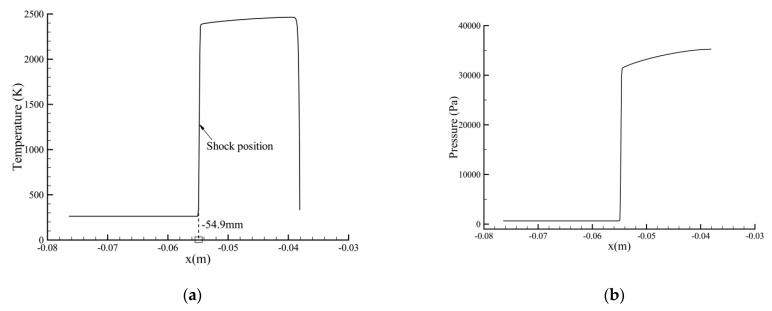
Computed distribution of (**a**) temperature and (**b**) pressure along the symmetry line.

Figure 2 shows the computed distribution of temperature along the symmetry line. A typical aerothermal phenomenon in hypersonic flows around a blunt body is observed. The free-stream temperature first undergoes a rapid increase across the shock, and then drops suddenly from over 2000 K to the fixed wall temperature within a thin thermal boundary layer. It is also found that the thicknesses of the shock and boundary layer are favorably thin, indicating a high accuracy of the BGK scheme for capturing discontinuities. The predicted shock location (−54.9 mm) shows good agreement with those from Guo et al. [29] (−54.6 mm) and Zhang et al. [27] (−55.0 mm).

**Figure 3:**
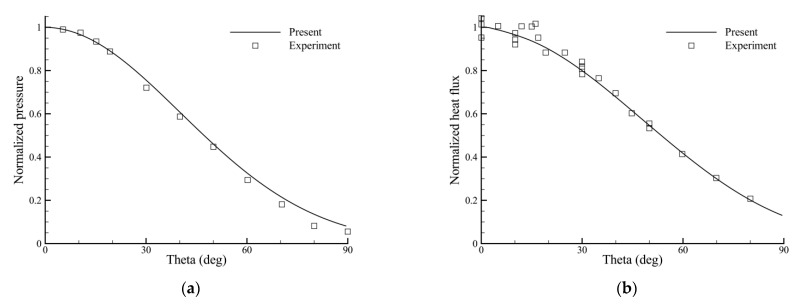
Distributions of (**a**) pressure and (**b**) heat flux along the cylinder wall.

The computed pressure and heat flux distributions along the cylinder wall are shown in Figure 3. The abscissa is defined as the angle from the stagnation point of the cylindrical body. Both the pressure and heat flux are normalized by their stagnation-point values, respectively, and those from the experiment are also presented. As shown in the figure, for both pressure and heat flux distributions, the present results agree very well with the experimental data.

**Figure 4:**
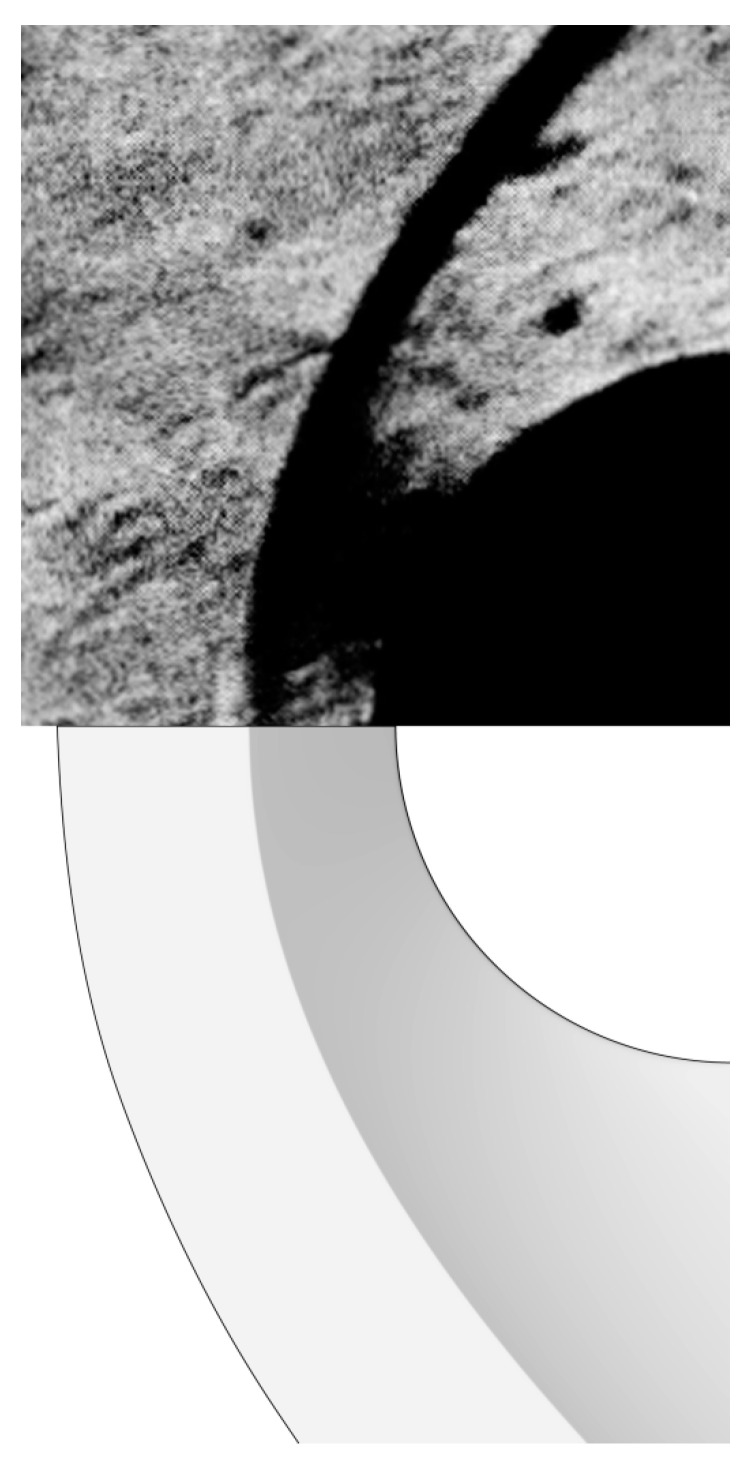
Computed density contour (**lower**) and Schlieren photograph (**upper**).

Finally, the computed density contour is compared to that from the Schlieren photograph [26]. As shown in Figure 4, good agreements were observed in the captured shock. All of the above results validate the satisfactory accuracy of the present BGK scheme for hypersonic flows.

## 4. Numerical Results and Discussions

In this section, two widely used thermal protection systems are investigated using the developed method. One is using the spike and the other one is using the opposing jet. Both their performances and mechanisms to reduce heat flux are analyzed.

### 4.1. Thermal Protection System by Using Spike

**Figure 5:**
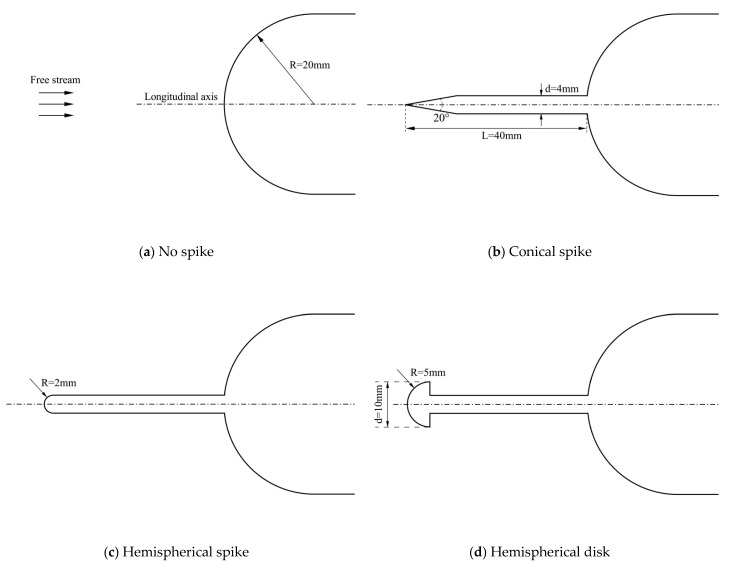
Different configurations of the spike attached to a hemispherical body.

The first case is a spiked blunt body experimentally investigated by Motoyama et al. [5]. The experiments are conducted in a 0.2 m radius Mach 7 hypersonic wind tunnel. The stagnation temperature is 860 K and the free-stream Reynolds number is 4 × 10^5^ (based on the diameter of the hemispherical body). Various configurations are tested in the experiment, and three typical models are considered in this case. One is a conical spike, the second is a hemispherical spike, and the third uses a hemispherical disk on a spike nose. The first two are often referred to as an aerospike model, while the third belongs to the aerodisk model. Their configurations are given in Figure 5, together with the original hemispherical body without a spike.

Because of the axis symmetry of geometry and flow conditions, the computation is simplified using a 2D axisymmetric model. Multi-block structured grids are employed to discretize the computational domain. The total number of grid cells used for these four models is about 0.12 million, which has been shown to be fine enough through a previous grid independence study. The first cell height to wall is set to keep $y^{+}$ near 1.

**Figure 6:**
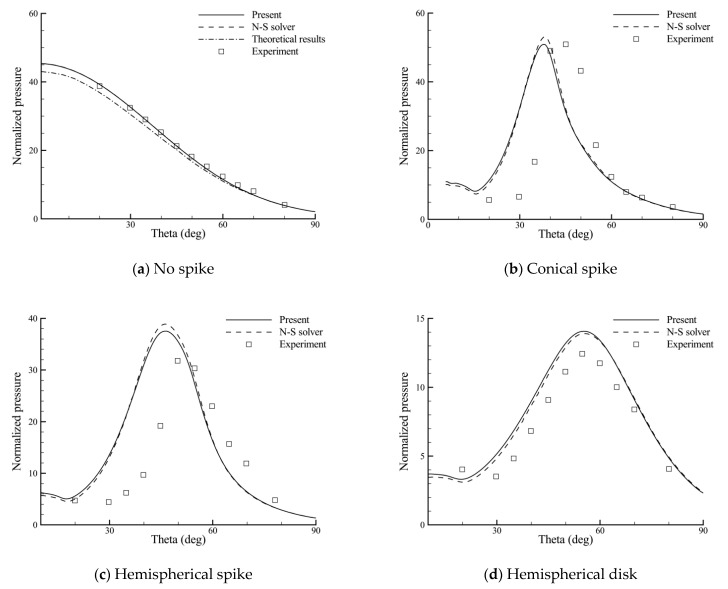
Surface pressure distributions of the hemispherical body with the use of different spikes.

Figure 6 presents the computed surface pressure distributions of the hemispherical body from the present method and a conventional N-S solver using the AUSM+ scheme. The abscissa represents the angle from the stagnation point of the hemisphere body, and the pressure data are normalized by the specific heat ratio and free-stream pressure. The experimental and theoretical results are also presented for comparison, if available. For all of the cases, both methods predict very similar pressure distributions. The numerical results of the no-spike case agree better with the experimental data than the inviscid theory [30]. This verifies the reliability of the BGK scheme for axisymmetric hypersonic flows. In the aerospike cases (conical spike and hemispherical spike), the predicted variations of pressure are, in general, consistent with the experimental data, despite the discrepancy in the shoulder location of the sharp pressure peak. As can be seen later, this region corresponds with the high heat flux region where reattachment of the shear layer occurs. In contrast, the change in pressure on the hemispherical body with the aerodisk (hemispherical disk) is relatively smooth, and, meanwhile, better agreements are found between the computed and experimental results.

**Figure 7:**
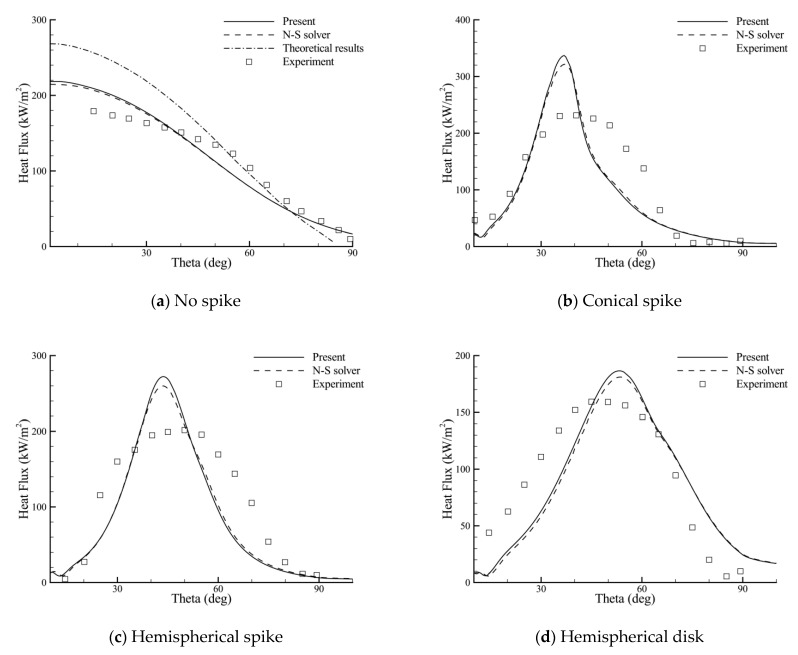
Surface heat flux distributions of the hemispherical body with the use of different spikes.

As a result of the lack of instantaneous surface temperature data in the original report, a uniform and constant wall temperature is assumed. Figure 7 shows the comparisons of the heat flux distributions for the same four cases, where the theoretical heat flux is obtained from the laminar flow theory [31]. Overall, the computed results are in line with the experimental trends, but the comparisons are less satisfactory. Particularly, in the aerospike cases, the predicted heat flux peaks appear earlier in the hemispherical body surface and seem higher and more sharp. A similar phenomena can also be found in references [32,33], and the deviations can be attributed to the assumption of a uniform wall temperature and experimental errors. In fact, it was argued in the original report [5] that “In the aerospike case, the heat flux distribution curve has a more-rounded peak than expected because of the estimated data around the peak in that case. For this reason, the actual heat flux at the shoulder of the body may be higher in the aerospike case”. Despite this, good agreements between the two methods indicate the effectiveness of the BGK scheme in the thermal protection analysis of the spike. As shown in the figure, the use of each spike can reduce the heat flux near the stagnation point of the hemispherical body. However, the heat fluxes at the shoulder of the hemispherical body are higher than the no-spike case, and the values may even exceed the stagnation-point value of the no-spike case, except for the hemispherical disk. Therefore, aerodynamic heating is more effectively reduced with use of an aerodisk.

**Figure 8:**
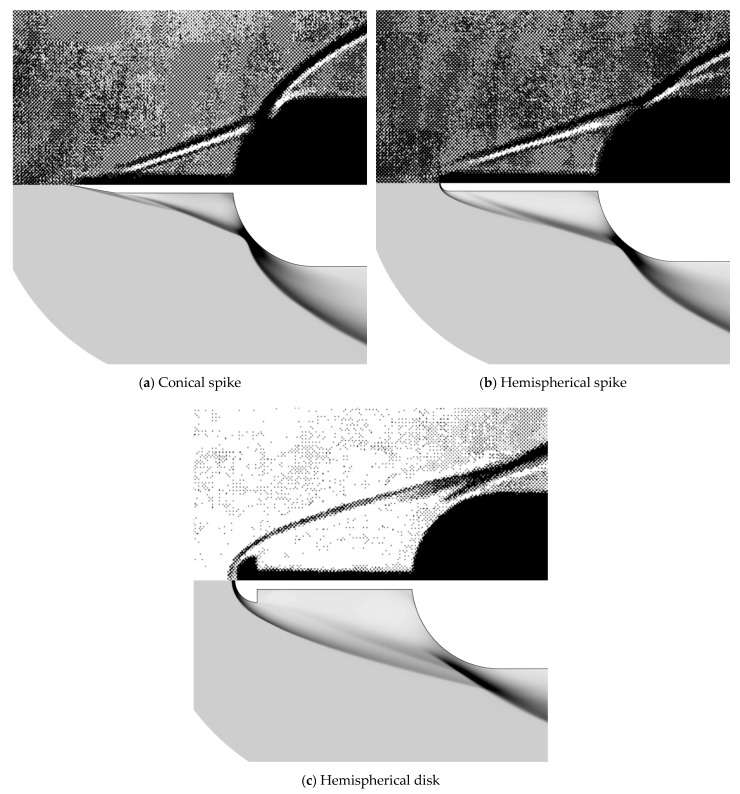
Density contours (**lower**) and Schlieren photographs (**upper**) for different spikes.

The above findings can be more intuitively seen in Figure 8. It is first observed that the computed density contours are close to the Schlieren photographs of the flowfield. In the aerospike cases, the thin shock wave from the spike nose, the shear layer from the near-wall separation, and the recompression shock from the reattachment point are clearly visible. However, the predicted location of the reattachment point is slightly ahead of the experimental measurement, which yields the errors in peak location shown in Figure 6 and Figure 7. In addition, a high temperature region can be observed around the reattachment point of the shear layer (which is not shown here). Then, in the aerodisk case, a bow shock is generated far from the hemispherical body. The body is mostly enveloped within the large recirculation region, which is separated from the inviscid flow within the bow shock by the flow separation that caused a shear layer. The captured reattachment shock agrees well with the Schlieren photograph in terms of both the location and structure. Furthermore, as the oblique shock does not directly impinge on the body surface, the temperature rise is not as significant as that in the aerospike cases.

### 4.2. Thermal Protection System by Using Opposing Jet

The second case is a blunt body experimentally studied by Hayashi et al. [34] in a blowdown-type supersonic wind tunnel. The blunt body has a diameter of 50 mm and the nozzle exit has a diameter of 4 mm. The free-stream conditions include Mach number $M_{\infty }=3.98$, total pressure *p*_0_ = 1.37 MPa, and total temperature *T*_0_ = 397 K. A uniform wall temperature *T_w_* = 295 K is assumed. An opposing jet is blown with a coolant gas at a total temperature *T*_0*j*_ = 300 K and specified total pressure ratio PR. The jet Mach number is 1.0, and the Reynolds number is 2.1 × 10^6^ (based on the diameter of the blunt body). The total pressure ratio PR is defined as the ratio of the total pressure of jet $p_{0j}$ to the total pressure of the free stream $p_{0\infty }$, i.e.,

$$PR=\frac{p_{0j}}{p_{0\infty }}$$

**Figure 9:**
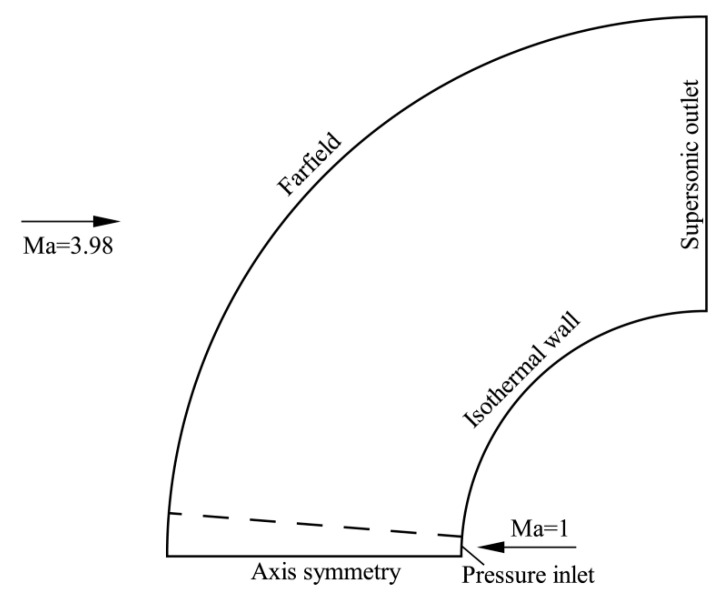
Computational model for the hypersonic flow past a blunt body with an opposing jet.

Four stable jet conditions of *PR* = 0, 0.4, 0.6, 0.8 are studied, where PR=0 represents no jet. The computational model for this case is shown in Figure 9. The computational grid used has a size of 401 × 301 and the first cell height to wall is Δ*s* = 1 × 10^−6^ m. The grid is tangentially clustered near the nozzle exit. The physical properties at the nozzle exit are determined by using the isentropic relations together with the prescribed total pressure ratio and total temperature.

Before showing the computed results, it is worth mentioning that a slight self-induced oscillation phenomenon is found in the flow fields of the opposing jet. Fortunately, these oscillations are small and become weakened with the increase in the total pressure ratio, and thus the results are evaluated by some averaging.

**Figure 10:**
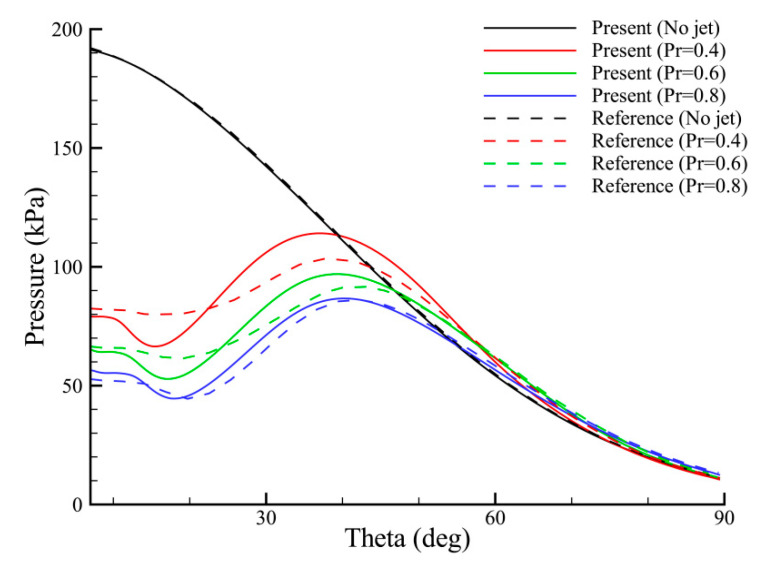
Surface pressure distributions for different total pressure ratios.

Figure 10 compares the computed surface pressure distributions with the numerical results of Hayashi et al. [35]. The horizontal axis is the angle from the stagnation point of the blunt body. For the no-jet case, both methods predict almost the same results. For the cases where the opposing jet blows, the overall pressure significantly decreases due to the presence of flow recirculation. In these cases, good agreements are observed over the majority of the curves, although the present results predict higher peaks and lower valleys near the recirculation region for *PR* = 0.4 and 0.6. It is also found that as PR increases, the pressure in the recirculation region gradually decreases.

**Figure 11:**
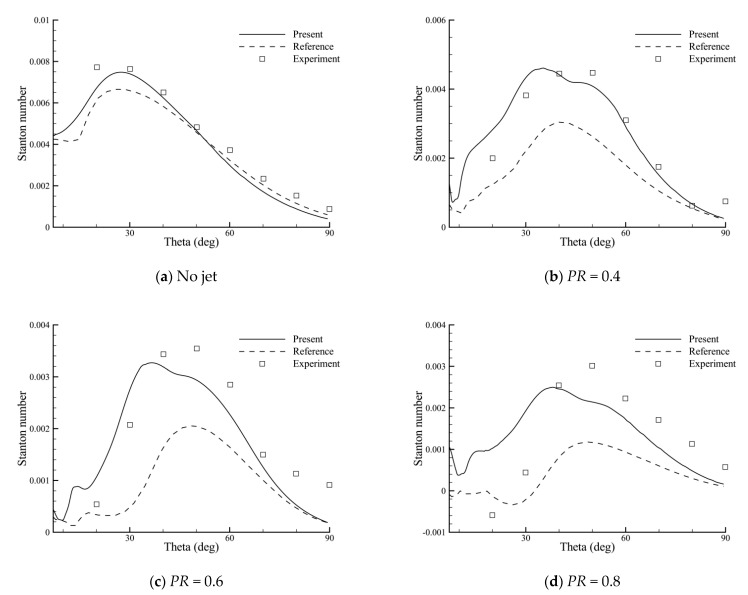
Surface heat flux distributions for different total pressure ratios.

The comparisons of the heat flux distributions for different total pressure ratios are shown in Figure 11. As in the experiments, the Stanton number St is used to compare each heat flux distribution, which is defined as

$$\begin{array}{l} St=\frac{q_{w}}{c_{p}(T_{aw}-T_{w})\rho_{\infty }V_{\infty }}\\ T_{aw}=T_{\infty }\left[1+\frac{\sqrt[{3}]{Pr}(\gamma -1)M_{\infty }^{2}}{2}\right] \end{array}$$

where $q_{w}$ is the heat flux, $T_{aw}$ is the adiabatic wall temperature, and $T_{w}$ is the wall temperature.

As shown in the figures, the heat flux in the no-jet case can be reduced using the opposing jet. With the increase in PR, this heat reduction is more effective. In the no-jet case, good agreements with experiment are obtained. In the cases of blowing the opposing jet, the values of heat flux are higher than the reference [35], but the peaks appear ahead. This is directly caused by the difference in the predicted recirculation regions. The discrepancy between the computed and experimental results is probably as a result of the surface roughness of the experimental model and the turbulence modeling error. Nevertheless, the present results show overall better agreements with the experiment, indicating the effectiveness of the BGK scheme in the thermal protection analysis of the opposing jet.

**Figure 12:**
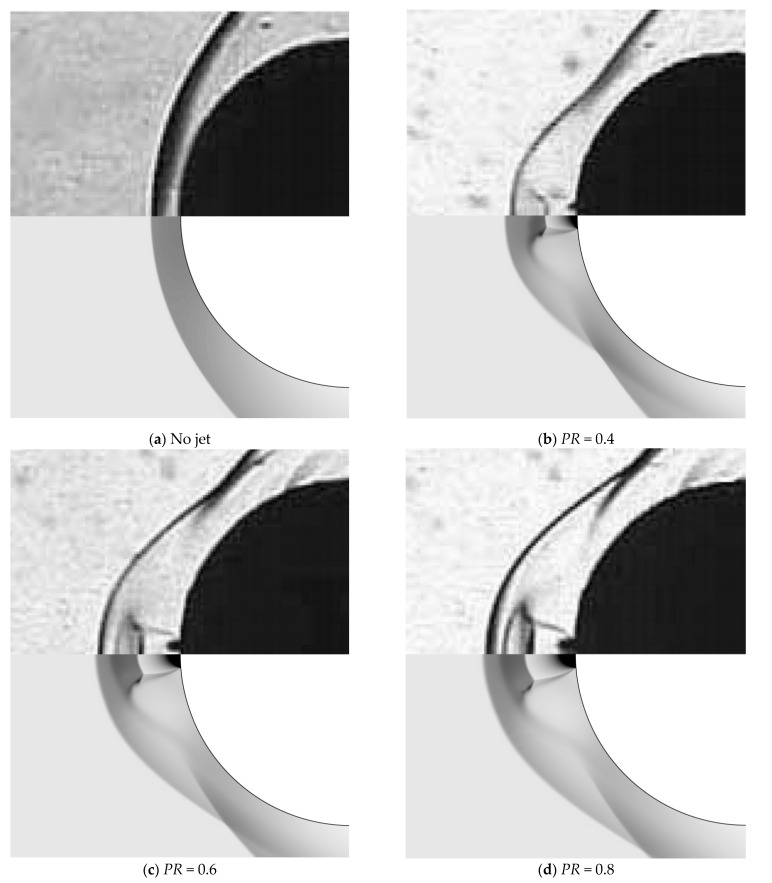
Density contours (**lower**) and Schlieren photographs (**upper**) for different total pressure ratios.

Figure 12 further shows the computed density contours and Schlieren photographs. In the no-jet case, the predicted bow shock agrees very well with that of the experiment, while in other cases, the position of the bow shock is located slightly upstream. This discrepancy is also seen in [35]. At each non-zero PR, a lot of unique characteristics are clearly and accurately captured by the present method, including Mach disk, contact surface, barrel shock wave, recompression shock wave, and the triple point. Particularly, the predicted Mach disk and barrel shock agree well with the experimental results.

**Figure 13:**
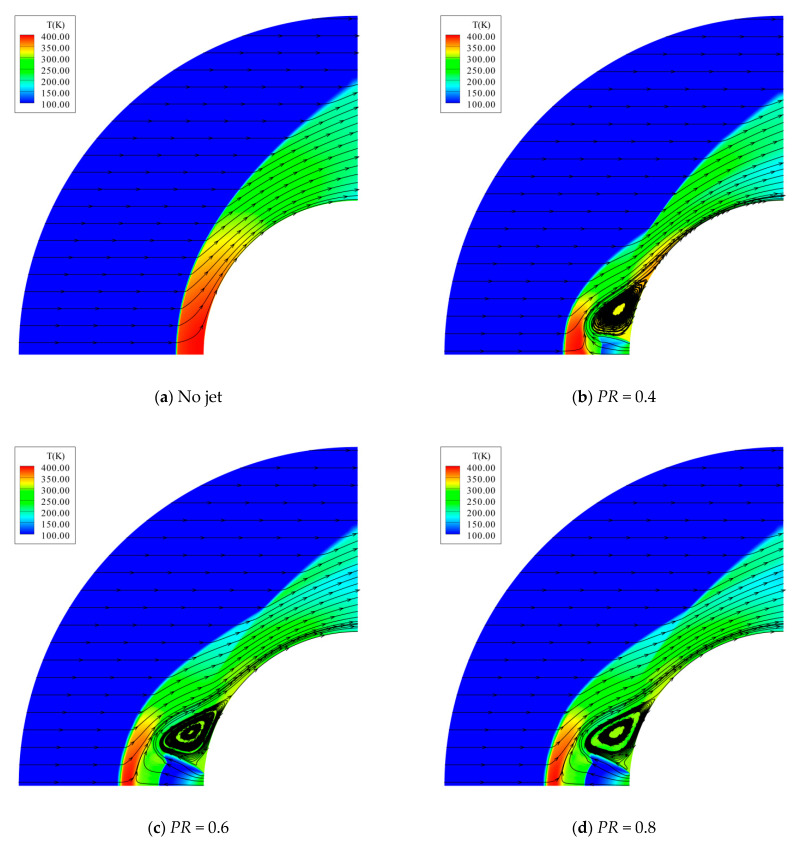
Temperature contours and streamlines for different total pressure ratios.

Finally, the computed temperature contours and streamlines are shown in Figure 13. From the visualizations of the flow fields and the curves of the heat flux distributions, it is found that the opposing jet reduces the heat flux mainly through two mechanisms: one is to prevent the hot flow downstream of the bow shock from reaching the body surface, and the other one is to form the recirculation region to protect the body surface. For the former one, the jet flow acts similarly to a mechanical spike. When it passes through the Mach disk and meets the free stream, the contact surface is formed. The contact surface moves upstream with an increase in the total pressure ratio. For the latter one, the recirculation region is formed around the nozzle exit and the recompression shock starts from the reattachment point. The jet flow passing through the barrel shock has a relatively low temperature, covering the recirculation region with cool gas. When the total pressure ratio increases, this cool flow region becomes larger and the temperature of the recirculation region decreases, resulting in a stronger cooling effect. This tendency is shown in Figure 12.

## 5. Conclusions

In recent years, the gas-kinetic BGK scheme has shown to be very promising in the simulation of hypersonic flows because of its advantages of having a delicate dissipation mechanism, automatic satisfaction of entropy condition, and positivity preserving. Motivated by this, it is herein extended to thermal protection system analysis, which is essential to vehicles at a hypersonic flight speed. Within a finite volume framework, the present BGK scheme is designed for the evaluation of the total fluxes across the cell interface by reconstructing the solution of the Boltzmann equation. A benchmark hypersonic flow past a cylindrical leading-edge model is first used to validate the developed code. Then, two representative thermal protection systems using spikes and opposing jets are investigated. In addition to predict the reduction of aerodynamic heating, different shapes of spikes and their effectiveness are analyzed in the former TPS and the effects of total pressure ratio of the jet are studied in the latter TPS. The computed results are compared with experimental, theoretical, or other numerical results. The mechanisms to reduce aerodynamic heating using two TPSs are also discussed. It is concluded that the BGK scheme shows good reliability and accuracy in thermal protection system analysis and has bright prospects in engineering applications.
